# Genetic Variability of 3′-Proximal Region of Genomes of Orf Viruses Isolated From Sheep and Wild Japanese Serows (*Capricornis crispus*) in Japan

**DOI:** 10.3389/fvets.2020.00188

**Published:** 2020-04-24

**Authors:** Kaori Shimizu, Asari Takaiwa, Shin-nosuke Takeshima, Ayaka Okada, Yasuo Inoshima

**Affiliations:** ^1^Laboratory of Food and Environmental Hygiene, Cooperative Department of Veterinary Medicine, Gifu University, Gifu, Japan; ^2^Department of Food and Nutrition, Jumonji University, Saitama, Japan; ^3^Education and Research Center for Food Animal Health, Gifu University (GeFAH), Gifu, Japan; ^4^The United Graduate School of Veterinary Sciences, Gifu University, Gifu, Japan; ^5^Joint Graduate School of Veterinary Sciences, Gifu University, Gifu, Japan

**Keywords:** genetic variability, Japanese serows, nucleotide sequence, orf virus, sheep

## Abstract

Orf virus is a prototype species of the genus *Parapoxvirus*, subfamily *Chordopoxvirinae*, family *Poxviridae*. Japanese orf viruses, infecting sheep and wild Japanese serows (*Capricornis crispus*), have been considered to be genetically closely related based on the sequence identities of the open reading frames (ORFs) 11, 20, and 132 in their genomes. However, since the genome size of orf viruses is about 140 kbp long, genetic variation among Japanese orf viruses remains unclear. In this study, we analyzed the sequences of ORFs 117, 119, 125, and 127 located in the 3′-proximal region of the viral genome using two strains from sheep and three strains from Japanese serows isolated from 1970 to 2007, and compared them with the corresponding sequences of reference orf viruses from other countries. Sequence analysis revealed that ORFs 125 and 127, which encode the inhibitor of apoptosis and viral interleukin (IL)-10, respectively, were highly conserved among the five Japanese orf viruses. However, high genetic variability with deletions or duplications was observed in ORFs 117 and 119, which encode granulocyte macrophage colony-stimulating factor and IL-2 inhibition factor (GIF), and inducer of cell apoptosis, respectively, in one strain from sheep and two strains from Japanese serows. Our results suggest that genetic variability exists in Japanese orf viruses even in the same host species. This is the first report of genetic variability of orf viruses in Japan.

## Introduction

Orf virus is a prototype species of the genus *Parapoxvirus*, subfamily *Chordopoxvirinae*, family *Poxviridae* (1). Orf virus has a linear double-stranded DNA genome (134–139 kbp) with high GC content (~63–64%) and encodes 132 putative gene products (2). Orf virus is the causative agent of orf disease, also known as contagious pustular dermatitis, contagious ecthyma, or scabby mouth mainly in sheep and goats, and can be transmitted to humans (3). In Japan, the first reports of orf virus infections in sheep and wild Japanese serows (*Capricornis crispus*) were published in 1952 (4) and 1979 (5), respectively. Previously, we have reported nucleotide sequence homology in three open reading frames (ORFs) 11, 20, and 132 among 13 orf viruses isolated or polymerase chain reaction (PCR)-detected from sheep and wild Japanese serows (6). These ORFs encode viral envelope (7), virus interferon resistance (8), and viral vascular endothelial growth factor (VEGF) (9), respectively. The amino acid sequences derived from ORFs 11 and 20 were identical among the 13 orf viruses, and only one amino acid substitution was found in ORF 132 in an orf virus isolated from sheep (6). Therefore, the three viral genes of Japanese orf viruses are highly conserved. However, since only a part of the whole genome (~140 kbp) has been sequenced so far, the degree of genetic variation in other regions remains unclear.

**Figure 1:**
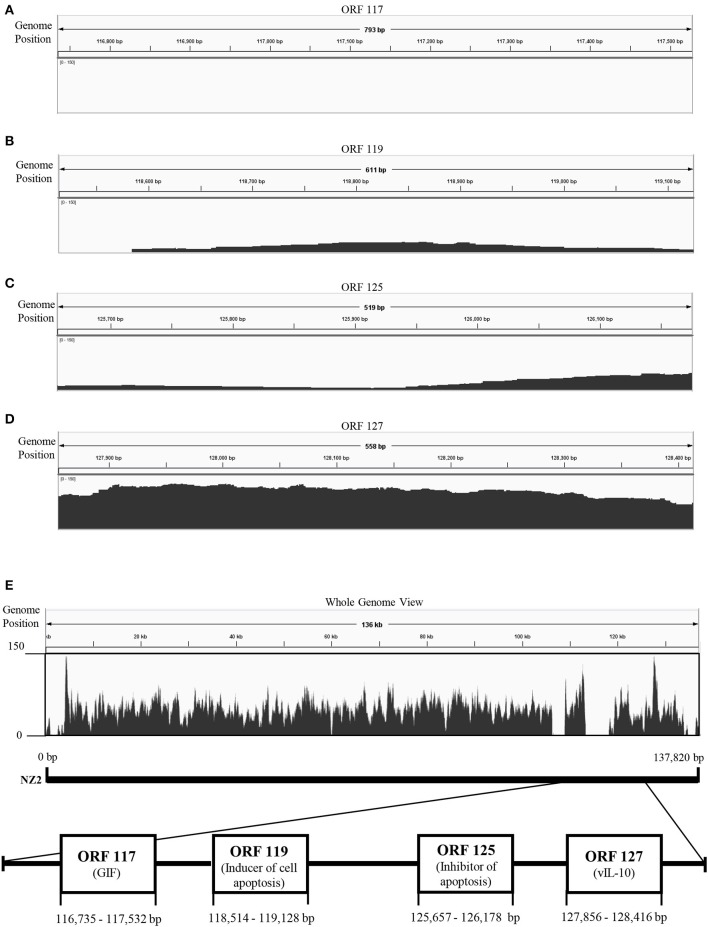
Visualization of next-generation sequencing (NGS) coverage of Japanese orf virus R-1 strain and the details of the open reading frame (ORF) analyzed in this study. NZ2 strain (GenBank accession no. DQ184476) was used as the reference strain. Binary version of the sequence alignment/map (bam) file was loaded onto the Integrated Genome Viewer (IGV) (10). The vertical axis shows the number of reads mapping to each location of the genome. Zoomed view of ORF 117 **(A)**, ORF 119 **(B)**, ORF 125 **(C)**, and ORF 127 **(D)**. Whole genome view **(E)**.

To explore genetic differences between Japanese orf viruses, we conducted next-generation sequencing (NGS) of some strains of these orf viruses. However, whole genome sequences were not obtained, due to the large number of unmapped reads in the 3′-proximal region of viral genome (Figure 1). We hypothesized that the 3′-proximal region of a viral genome has genetic variation. Thus, in the present study, we characterized four ORFs 117, 119, 125, and 127, which are located in the 3′-proximal region of the viral genome. ORFs 117, 119, 125, and 127 encode granulocyte macrophage colony-stimulating factor and interleukin 2 (IL-2) inhibition factor (GIF) (11), inducer of cell apoptosis (12), inhibitor of apoptosis (13, 14), and viral IL-10 (15), respectively.

## Materials and Methods

### Viruses

For epidemiologic and genetic characteristics of Japanese orf virus, five Japanese strains isolated from 1970 to 2007 were used. Two strains of Iwate (16) and HIS (17) were isolated from sheep and three strains of S-1 (18), R-1 (19), and GE (6) were isolated from wild Japanese serows (Table 1). Viruses were propagated in fetal lamb lung cells (kindly provided by Dr. H. Sentsui, Nihon University, Japan) at 37°C in Dulbecco's modified Eagle's medium (Wako, Osaka, Japan) supplemented with 10% fetal bovine serum (PAA Laboratories, Pasching, Austria).

**Table 1:** Japanese and reference orf viruses used in this study.

**Strain**	**Host**	**Year of isolation**	**Country**	**Deposited and reference accession no.^a^**				**References**
				**ORF 111–119**	**ORF 125**	**ORF 127**	**Complete genome**	
Iwate	Sheep	1970	Japan	LC487906	LC476578	LC476583		(16, 20, 21)
HIS	Sheep	2004	Japan	LC476574	LC476579	LC476584		(17, 21)
S-1	Japanese serow	1985	Japan	LC476575	LC476580	LC476585		(18, 20, 21)
R-1	Japanese serow	1999	Japan	LC476576	LC476581	LC476586		(19, 20)
GE	Japanese serow	2007	Japan	LC476577	LC476582	LC476587		(6)
NZ2	Sheep	1982	New Zealand				DQ184476	(2)
IA82	Sheep	1982	USA				AY386263	(22)
SA00	Goat	2000	USA				AY386264	(22)
NA1/11	Sheep	2011	China				KF234407	(23)
GO	Goat	2012	China				KP010354	(24)
YX	Goat	2012	China				KP010353	(24)

a *ORF, open reading frame*.

### Whole Genome Re-sequencing and Assembly

Total DNA extracted from virus-infected cells using a DNeasy Blood and Tissue Kit (Qiagen, Hilden, Germany) were used for constructing the libraries using Nextera XT DNA sample Prep Kit (Illumina, San Diego, CA, USA), and sequenced using an Illumina MiSeq (Illumina). Obtained short read sequences collected in FASTQ files were aligned to the orf virus strain NZ2 (DQ184476) as the reference genome using Burrows–Wheeler transformation (BWA) ver 0.7.12-r103 software (25) and constructed binary version of the sequence alignment/map (bam) file using SAM tools ver. 0.1.19-96b5f2294a software (26).

### Analysis of ORF

DNA was extracted from virus-infected cells using a DNeasy Blood and Tissue Kit (Qiagen) according to the manufacturer's instructions. Four different PCRs were carried out with GoTaq Hot Start Green Master Mix (Promega, Madison, WI, USA) using Veriti thermal cycler (Applied Biosystems, Foster City, CA, USA). The PCR conditions and analyzed ORFs are provided in Supplementary Table S1 and Figure 1, respectively. PCR products were purified using NucleoSpin Gel and PCR Clean-up (Macherey-Nagel, Duren, Germany), and the nucleotide sequences were determined by direct sequencing using a BigDye Terminator Cycle Sequencing Kit v3.1 (Applied Biosystems, Foster City, CA, USA). Sequence analysis was carried out using the software Genetyx-Win version 13 (Genetyx, Tokyo, Japan), and phylogenetic analysis was performed using the MEGA7 program (27). Phylogenetic trees were constructed using maximum likelihood methods, and the reliability of the branches was evaluated by 1,000 replicates. Sequence and phylogenetic analyses were compared with the reference orf viruses (Table 1).

## Results

NGS was performed using total DNA extracted from the concentrated virus. However, whole genome sequences were not obtained, possibly due to the large number of deletion in the 3′-proximal region of viral genome (Figure 1).

**Figure 2:**
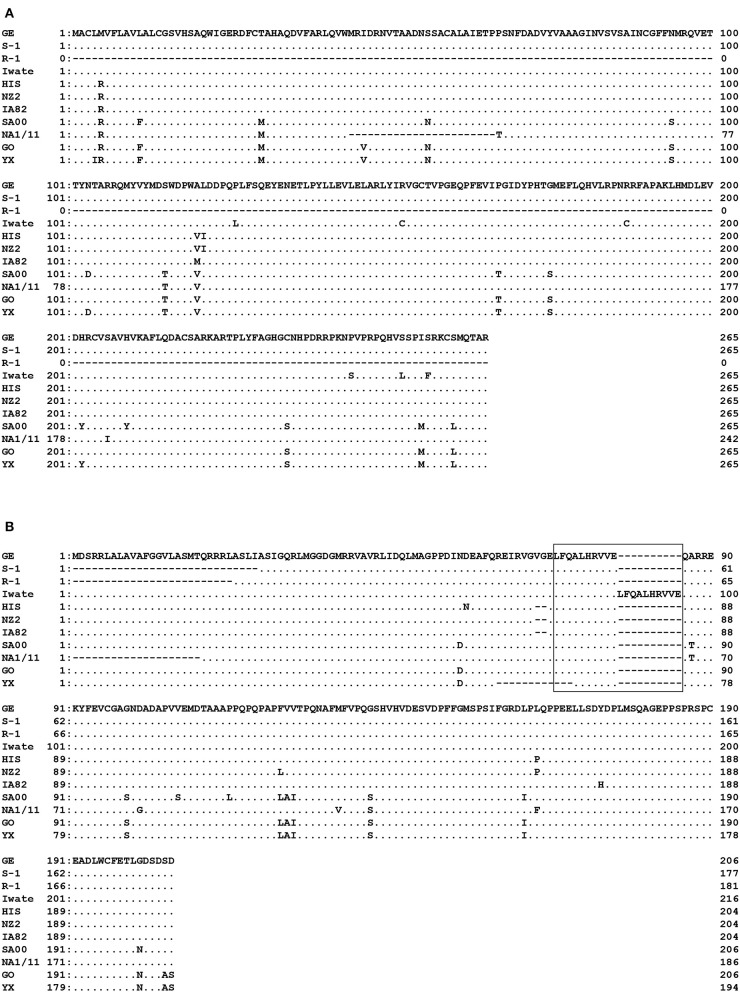
Alignment of the amino acid sequences derived from ORF 117 **(A)** and ORF 119 **(B)**. Amino acids identical to GE strain at the given positions are represented by dots. R-1 strain completely lacked ORF 117. In ORF 119, deletions were observed in the first half of the nucleotide sequences in S-1 and R-1 strains. In the box, duplicate in Iwate strain is shown.

Specific PCR products of 799 bp for ORF 117 and 537–652 bp for ORF 119 were obtained from the four strains (Iwate, HIS, S-1, and GE) and from all five Japanese orf viruses, respectively. High genomic variability was seen in ORFs 117 and 119 in Japanese orf viruses. In ORF 117, 96.6–100% nucleotide identity was observed among four strains. Surprisingly, R-1 strain from Japanese serow completely lacked ORF 117 (Figure 2A). Partial deletion in ORF 117 was also observed in the amino acid sequences in reference orf virus strain NA1/11 isolated from sheep in China. In ORF 119, deletions were observed in the first half of the amino acid sequences in S-1 and R-1 strains as well as the reference Chinese NA1/11 strain (Figure 2B). Two and 12 amino acid deletion was observed in HIS and three reference strains from sheep and goat (NZ2, IA82, and YX), respectively. In the Iwate strain, 10 amino acids were found to be duplicated.

**Figure 3:**
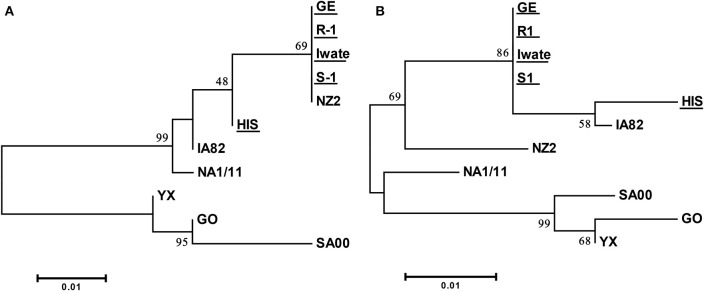
Phylogenetic tree based on deduced amino acid sequences derived from ORF 125 **(A)** and ORF 127 **(B)**. Strains used in this study are underlined. The percentage bootstrap values calculated from 1,000 replicates are indicated above the internal nodes.

Specific PCR products of 522 and 561 bp were obtained for ORFs 125 and 127 from all of five Japanese orf viruses. Amino acid sequences derived from these ORFs from four Japanese orf viruses (Iwate, S-1, R-1, and GE) were found to be 100% identical. The sequence from HIS strain revealed only two and seven amino acid substitutions in ORFs 125 and 127, respectively (Supplementary Tables S2, S3). The sequences of ORFs 125 and 127 were highly conserved among Japanese orf viruses. In the phylogenetic analysis, there were mainly two branches, and all Japanese orf viruses were classified into the same group (Figure 3). Our results indicate that Japanese orf viruses are closer to the IA82 and NZ2 strains isolated in the United States and New Zealand, respectively, than other reference strains. Sequences obtained in this study were submitted to DDBJ/EMBL/GenBank, and the accession numbers are given in Table 1.

## Discussion

In this study, we carried out ORF sequence analysis for five Japanese orf viruses, and our results revealed that the sequences of ORFs 125 and 127 were highly conserved. However, high genomic variability was seen in ORFs 117 and 119. Observed genetic variability was found to be the 3′-proximal region of Japanese orf viruses. To the best of our knowledge, this is the first report on the genetic variability of Japanese orf viruses.

In the phylogenetic analysis of ORF 125, Japanese orf viruses isolated from Japanese serows were classified into a group isolated from sheep. It has been reported that analyses of the phylogenetic tree of 47 ORFs including ORF 125 were found to assist in easily distinguishing between goat- and sheep-originated orf viruses (24). These results indicate a possibility that sheep orf virus may have infected Japanese serows. Furthermore, analyses of the phylogenetic tree of ORFs 125 and 127 clearly showed that the Japanese orf viruses were closer to IA82 and NZ2 strains than to other reference strains. In Japan, sheep are frequently imported from the United States and New Zealand for improved growth and to encourage breeding (28). Therefore, it is possible that these orf viruses came along with the imported animals and were introduced into breeding sheep and wild Japanese serows in Japan.

Our results showed genetic variability in ORFs 117 and 119 in the Japanese orf viruses, suggesting that there is heterogeneity even in viruses infected with the same host species. In addition, deletions in ORF 119 were observed in Japanese (HIS, S-1, and R-1) and reference (NZ2, IA82, NA1/11, and YX) strains. Based on the previous comparative analysis, it is presumed that genes in the central region of the orf virus genome are more conserved, whereas those in the terminal region show remarkably high variability (29). Notably, this variability is accompanied by a high frequency of gene recombination and nucleotide deletions (23). The genetic analysis of ORFs 117 and 119 may help to characterize or differentiate strains that are otherwise shown to be identical by the envelope coding genes (30). A previous study demonstrated that viruses with high deletion in ORFs 114–120 showed low virulence in animal inoculation experiments and that genomic deletions attenuate virulence (24). At present, the relationship between the deletion of ORF 117 and virulence in the R-1 strain is unknown. Therefore, there is a need to analyze the correlation between genetic variability and virulence in more detail.

In this study, it was revealed that there were differences in conservation and variability among ORFs. Viral IL-10 encoded by ORF 127 shares remarkable similarity to mammalian IL-10. Mammalian IL-10 is highly conserved across all mammalian species (15). IL-10 is a multifunctional cytokine that has suppressive effects on inflammation, antiviral responses. Orf virus produces viral IL-10 by itself and avoids host's inflammatory and immune response by it (31). This suggests that viral IL-10 encoded by ORF 127 might require high conservation in orf virus. On the other hand, GIF encoded by ORF 117 does not resemble any known mammalian granulocyte-macrophage colony–stimulating factor (GM-CSF)- or IL-2-binding proteins, and indeed, there are no reports of any other protein capable of binding both GM-CSF and IL-2. In addition, human GM-CSF does not respond in sheep cells due to its inability to bind to ovine receptor (32). Therefore, GIF was thought to have evolved a unique binding specificity in sheep, the natural host of the orf virus (33). This suggests that GIF encoded by ORF 117 is gene whose necessity changes depending on the host species. It is thought that differences in necessity of gene may affect conservation and variability of the gene encoded by ORFs.

Japanese serows are wild animals and a natural monument that is endemic in Japan (34). Japanese serows are often witnessed in mountain villages and can come into contact with livestock sheep. There have been reports that a single strain of orf virus caused outbreak of proliferative dermatitis in various ruminant species at a zoo (35). Orf virus from Japanese serows can be spread to sheep or farmers, or orf virus from sheep can be spread to Japanese serows. It is important to know the characteristics of Japanese orf viruses in order to reduce the spread risk.

We tried NGS analysis, but it was unsuccessful. NGS results indicated the 3′-proximal region of the genome of Japanese orf viruses has genetic variation. Our results obtained by Sanger sequencing for variable region of Japanese orf viruses may be useful for understanding the characteristics of these viruses. However, we analyzed the limited region of the viral genomes, and sequencing other regions using improved methods for NGS might be required to better understand the characteristics of Japanese orf viruses.

## Data Availability Statement

Sequence data obtained in this study is available in the DDBJ/EMBL/GenBank (accession nos. LC476574–LC476587 and LC487906).

## Author Contributions

KS and YI analyzed all data and were major contributors in writing the manuscript. KS, AT, ST, and AO performed the nucleotide/amino acid sequencing and phylogenetic analysis. All authors read and approved the final manuscript.

## Conflict of Interest

The authors declare that the research was conducted in the absence of any commercial or financial relationships that could be construed as a potential conflict of interest.

## Abbreviations

GIF: granulocyte-macrophage colony-stimulating factor and IL-2 inhibition factor
IL-2: interleukin 2
ORF: open reading frame
PCR: polymerase chain reaction
VEGF: vascular endothelial growth factor.
